# Editorial: International Perspectives on Health and Safety among Dairy Workers: Challenges, Solutions and the Future

**DOI:** 10.3389/fpubh.2017.00294

**Published:** 2017-11-17

**Authors:** Martina Carola Jakob, John Rosecrance

**Affiliations:** ^1^Leibniz Institute for Agricultural Engineering Potsdam-Bornim (LG), Potsdam, Germany; ^2^College of Veterinary Medicine and Biomedical Sciences, Colorado State University, Fort Collins, CO, United States

**Keywords:** dairy, milking parlor, workload assessment, parlor worker, risk control

**Editorial on the Research Topic**

**International Perspectives on Health and Safety among Dairy Workers: Challenges, Solutions and the Future**

The purpose of this special topics edition of Frontiers in Public Health was to present an international perspective on current occupational health research related to workers in the dairy industry. The 32 contributing authors were occupational health researchers from eight countries, including Argentina, Finland, Ireland, Italy, Spain, Sweden, Uganda, and the United States. The majority of authors were part of the International Dairy Research Consortium, a group of international dairy researchers focused on improving the health and safety of dairy workers throughout the world. In the first year of publication, there were approximately 16,000 views and 1,800 article downloads of the 10 published papers.

Milking cows is one of the major work tasks on dairy farms regardless of herd size. The occupational risks associated with milking cows are just as significant for the Latino worker (Menger et al.; Menger et al.) in a large herd American dairy as they are for the Ugandan farmer (Lunner-Kolstrup and Ssali) with five dairy cows. The perception of milk production by consumers in developed countries is an industry that is primarily automated. Although milking tasks have changed in modern milking parlors, parlor workers still experience work-related aches and pain and have more accidents compared to other professions (Pinzke). In this issue, researchers present comprehensive workload analyses and reported that preparing the udder and attaching the milking cluster is associated with awkward postures and high muscular loads of the upper-limb among dairy workers (Mixco et al.; Masci et al.). Other than robotic milking (Karttunen et al.), there are few mechanical interventions that completely eliminate the physical work exposures that are associated with musculoskeletal disorders among dairy parlor workers. Occupational challenges, such as awkward working postures, repetitive tasks, long or unfavorable working hours, cold or hot and wet working environments, significant time pressures, and high workloads do little to attract the next generation to take up this profession.

There has been a worldwide trend of increasing farm size with a simultaneous reduction in the number of farms (Pinzke). Increasing farm size requires hiring more workers, often in regions where the only labor source available are immigrant laborers. Additionally, farm owners need to learn how to manage and train a very diverse workforce in farm operations, including health and safety. Among the published articles, four address the issue regarding health and safety and worker training. Menger et al. and Menger et al. state in their conclusions that the management has a large impact on worker perception of health and safety. Managements’ leadership skills are of major importance. Clear communication of tasks, addressing health and safety, providing adequate tools, or giving feedback reduces frustration and increases job satisfaction. Rovai et al. present the development of a unique series of short weekly toolbox talks to train immigrant dairy workers on issues related to animal care, cow comfort, and personal safety. The outcomes resulting from the dairy toolbox talks included increased knowledge, greater safety awareness, and enhanced job satisfaction for the workers. Finally, Menger et al. and Menger et al. suggest that the development of dairy training programs emphasize and consider the cultural uniqueness of the target population. Cultural specification of training is a significant issue as the majority of dairy workers in developed countries are immigrants. This issue is also emphasized by Lunner-Kolstrup and Ssali who investigated a very different situation of small-scale dairy farmers in Uganda. Additionally, Furey et al. addressed the role of financial threats on the mental wellbeing of dairy farmers. This is an important issue because of increasing farm sizes and new, highly sophisticated technologies that require significant resources, while both are occurring in an economy of volatile milk prices.

Although requiring significant financial investment, one of the most effective measures of reducing the workload on a dairy farm is investing in automatic milking systems (milking robots). Karttunen et al. describe the health and safety situation of Finnish farms with milking robots. A robot can reduce labor and musculoskeletal risk, but also creates new stresses and challenges for the farmer. Finally, Manbeck et al. discuss the dangers of on-farm manure storage pits that contain both toxic and asphyxiating gases such as hydrogen sulfide, carbon dioxide, methane, and ammonia. The authors present online design aids to evaluate manure pit ventilation systems that would reduce entry risk.

## Author Contributions

Both authors contributed equally.

## Conflict of Interest Statement

The authors declare that the research was conducted in the absence of any commercial or financial relationships that could be construed as a potential conflict of interest.

